# Dual transcriptional inhibition of glutamate and alanine racemase is synergistic in Mycobacterium tuberculosis

**DOI:** 10.1099/mic.0.001484

**Published:** 2024-08-08

**Keywords:** ADCL, Alr, cell wall precursors, CRISPRi, glutamate racemase, peptidoglycan synthesis

## Abstract

Synergistic interactions between chemical inhibitors, whilst informative, can be difficult to interpret, as chemical inhibitors can often have multiple targets, many of which can be unknown. Here, using multiplexed transcriptional repression, we have validated that the simultaneous repression of glutamate racemase and alanine racemase has a synergistic interaction in *Mycobacterium tuberculosis*. This confirms prior observations from chemical interaction studies and highlights the potential of targeting multiple enzymes involved in mycobacterial cell wall synthesis.

## Introduction

**Fig. 1.**
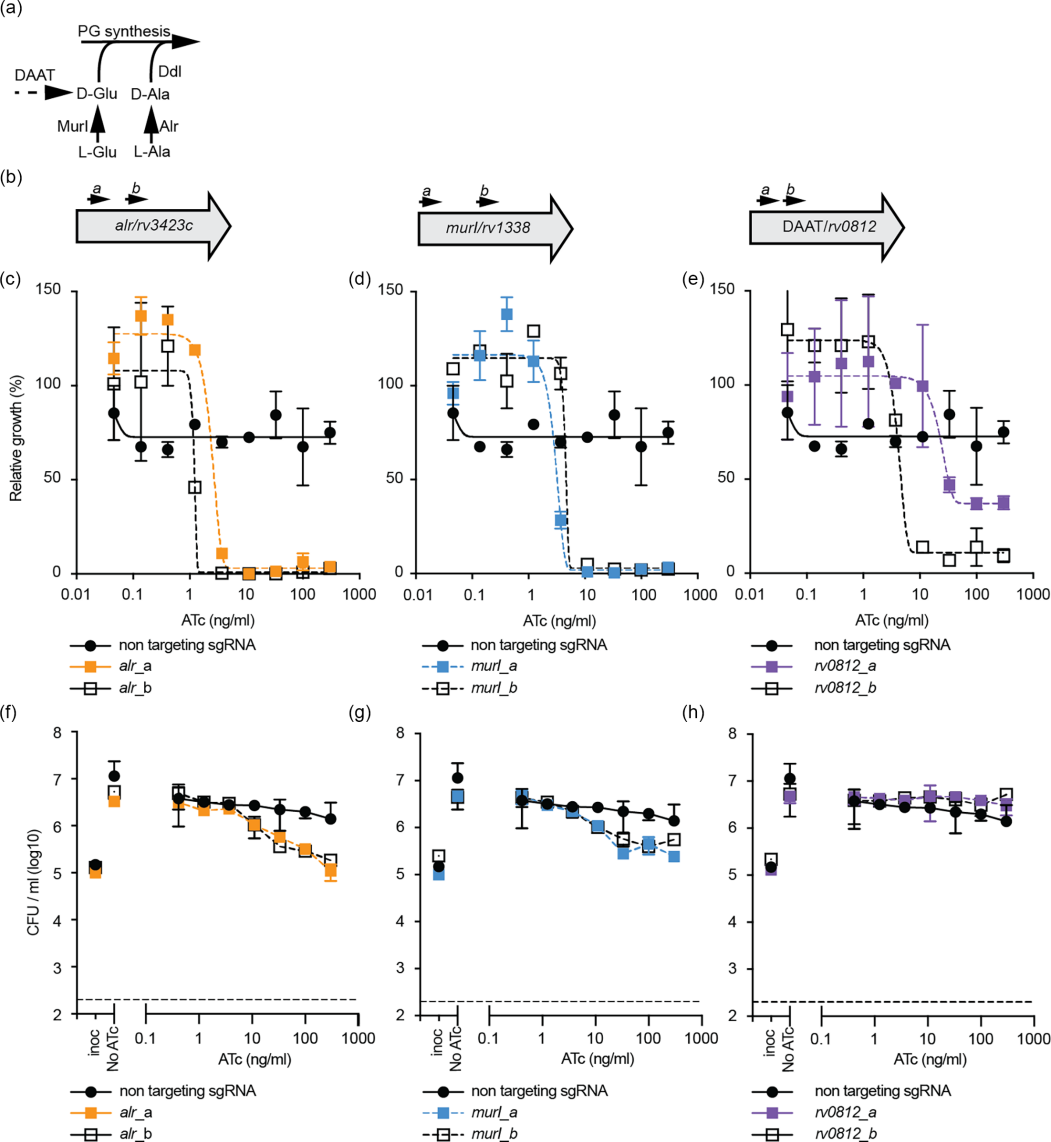
Transcriptional repression of *alr*, *murI* and *rv0812* in *M. tuberculosis*. (**a**) Schematic of enzymatic reactions catalysed by MurI, Alr, Ddl and DAAT (if present). (**b**) Approximate binding locations of gRNA sequences targeting each gene. a and b correspond to gRNA_a and gRNA_b. Sequences and relevant gRNA information is contained within Table 1. (**c–e**) Growth of *M. tuberculosis* expressing either a non-targeting or (**c**) *alr*, (**d**) *murI* or (**e**) *rv0812* targeting gRNA with differing levels of ATc (anhydrotetracycline). The non-targeting sequence (CGAGACGCATTAATCGTCTCC) is the nucleotide sequence between the *bsmB1* cloning sites in pJR965. Cultures were grown in 96-well plates starting from an OD_600_ of 0.005. OD_600_ was measured on day 10. Results are the mean±range of two replicates from a representative experiment and expressed relative to a no ATc control. (**f–h**) Viability of *M. tuberculosis* expressing either a non-targeting or (**f**) *alr*, (**g**) *murI* or (**h**) *rv0812* targeting gRNA with differing levels of ATc. Cultures were grown in 96-well plates starting from an OD_600_ of 0.005. c.f.u. ml^−1^ was determined after 5 days of growth and is presented as the mean±range of two replicates. Inoc, inoculating c.f.u. ml^−1^ on day 0.

*Mycobacterium tuberculosis* remains a leading cause of death by infectious disease [1]. The mycobacterial cell wall is an interconnected structure of peptidoglycan (PG), arabinogalactan and mycolic acids that generates an impermeable barrier to resist host defences and limits the uptake of many antibiotics [2]. Many clinically utilized antibiotics, including the front-line antibiotic isoniazid, target the mycobacterial cell wall highlighting the essentiality and druggability of this process. Inhibiting cell wall synthesis can also increase cell permeability and synergize with functionally unrelated antibiotics by increasing their uptake [3]. d-Cycloserine (DCS) is a clinically utilized antibiotic that inhibits d-alanine:d-alanine ligase (Ddl) and alanine racemase (Alr), both of which contribute to mycobacterial PG synthesis (Fig. 1a) [4,8]. DCS synergizes with other drugs that target various components of PG synthesis including β-chloro-d-alanine (BCDA) that targets both glutamate racemase (MurI), an enzyme that contributes d-glutamate to PG synthesis (Fig. 1a) [5,9], and Alr, which contributes d-alanine [9,11]. Currently, DCS is used as a second-line agent primarily in the treatment of mycobacterial drug resistance, whilst BCDA, which is not approved for clinical use, has been well studied in mycobacteria [5,9].

**Table 1.** gRNA used in this study

gRNA name	Target gene	Target sequence [coding (5′−3′)]	PAM (non-coding 5′−3′, NN…)	PAM score*	sgRNA length (bp)	Fwd oligo (5–3′) (GGGA_)	Oligo rev sequence (AAAC_)
alr_a	Rv3423c	GAGAATGTCGGAAAGCCAAAC	CCAGAAC	5	21	GGGAGTTTGGCTTTCCGACATTCTC	AAACGAGAATGTCGGAAAGCCAAAC
alr_b	Rv3423c	CGCAGGCCCGCGAACAAGGGGT	CCAGAAA	3	22	GGGAACCCCTTGTTCGCGGGCCTGCG	AAACCGCAGGCCCGCGAACAAGGGGT
murI_a	Rv1338	CGTCGGGGGACTGACGGTCGC	CCGGAAT	12	21	GGGAGCGACCGTCAGTCCCCCGACG	AAACCGTCGGGGGACTGACGGTCGC
murI_b	Rv1338	CGAGCGCTACCAGGTGCCCGT	CGAGCAT	7	21	GGGAACGGGCACCTGGTAGCGCTCG	AAACCGAGCGCTACCAGGTGCCCGT
rv0812_a	Rv0812	AGCCGGGTATGCCGCTGCTGC	GCAGGAT	9	21	GGGAGCAGCAGCGGCATACCCGGCT	AAACAGCCGGGTATGCCGCTGCTGC
rv0812_a	Rv0812	AACCGGATCTCCCCAGGTGGC	CGGGAAG	4	21	GGGAGCCACCTGGGGAGATCCGGTT	AAACAACCGGATCTCCCCAGGTGGC

* See Rock *et al*. [18]. Nature Microbiology

Whilst these prior results suggest that simultaneous inhibition of Alr and MurI would produce a synergistic drug combination, there is no experimental validation of this interaction as both DCS and BCDA are able to inhibit or interact with multiple targets [4,5, 7, 12,15]. Furthermore, the work in *M. smegmatis* has demonstrated that an aminodeoxychorismate lyase (ADCL) (MSMEG_5795) has significant d-amino acid transaminase moonlighting activity that can, in overexpressing strains, compensate for the loss of MurI by providing an alternative source of d-glutamate [16]. Here, using CRISPR interference (CRISPRi) transcriptional repression, the aim of this study was to (i) validate the synergistic interaction between the inhibition of *alr* and *murI* and (ii) determine if the ADCL homologue in *M. tuberculosis* (*rv0812*) has sufficient d-amino acid transaminase activity to compensate for the inhibition of *murI* expression [17]. Specifically, this was achieved by investigating the consequences of transcriptionally inhibiting *alr*, *murI* and *rv0812* alone and in combination.

## Methods

Mycobacterial CRISPRi uses 20–25 nucleotide guide RNA (gRNA) sequences to guide deactivated Cas9 (dCas) to target genes, where dCas9 binds to sterically inhibit transcription [18]. We individually cloned two unique gRNA sequences with complementarity to the non-template sequence of each gene into separate CRISPRi plasmids (i.e. pLJR965) using previously published protocols [19,22] (Fig. 1b). Sequence-verified CRISPRi plasmids were electroporated into *M. tuberculosis* strain, mc^2^6230 (∆*panCD*, ∆RD1), as previously described. mc^2^6230 is a BSL2 avirulent auxotroph that has been approved for use under BSL2 containment at the University of Otago. The current literature is consistent with the mc^2^6230 strain, supplemented as required for *in vitro* use, not having effects on cell wall biosynthesis. Reduced phthiocerol dimycocerosate (PDIM) content has been noted in this strain, but this has also been found in other laboratory-propagated strains of *M. tuberculosis* [23]. Using 96-well plate growth assays, we initially investigated the consequences of repressing *alr*, *murI* and *rv0812* alone. Assays were performed as previously described [19,21, 22]. Briefly, *M. tuberculosis* strains containing gRNA that targeted genes of interest were inoculated into 96-well plates containing 7H9 supplemented media [i.e. with OADC (0.005 % oleic acid, 0.5 % BSA, 0.2 % dextrose and 0.085 % catalase), 0.05 % tyloxapol (Sigma), 25 µg ml^−1^ pantothenic acid and 25 µg ml^−1^ kanamycin], with a threefold dilution of ATc at a starting OD_600_ of 0.005 in a total volume of 150 µl. Ninety-six-well plates were incubated without shaking at 37 °C for 10 days, and OD_600_ was measured in a Varioskan LUX microplate reader. To determine the effects on cell viability, plates were prepared as mentioned earlier, and the culture was extracted from wells containing relevant ATc concentrations on day 5, diluted and then spotted onto supplemented 7H11. Colonies were counted after 4 weeks at 37 °C.

## Results and discussion

Consistent with prior reports of gene essentiality, gRNA targeting *alr* and *murI* inhibited growth but had a bacteriostatic phenotype and did not reduce viability compared to the starting inoculum [21,24, 25] (Fig. 1c, d, f, g). Both gRNAs targeting *rv0812* impaired the growth of *M. tuberculosis*, with no effect on viability (Fig. 1e, h). This impaired growth is in agreement with prior Tnseq experiments showing that the disruption of *rv0812* had a growth defect in *M. tuberculosis* H37Rv [26].

**Fig. 2.**
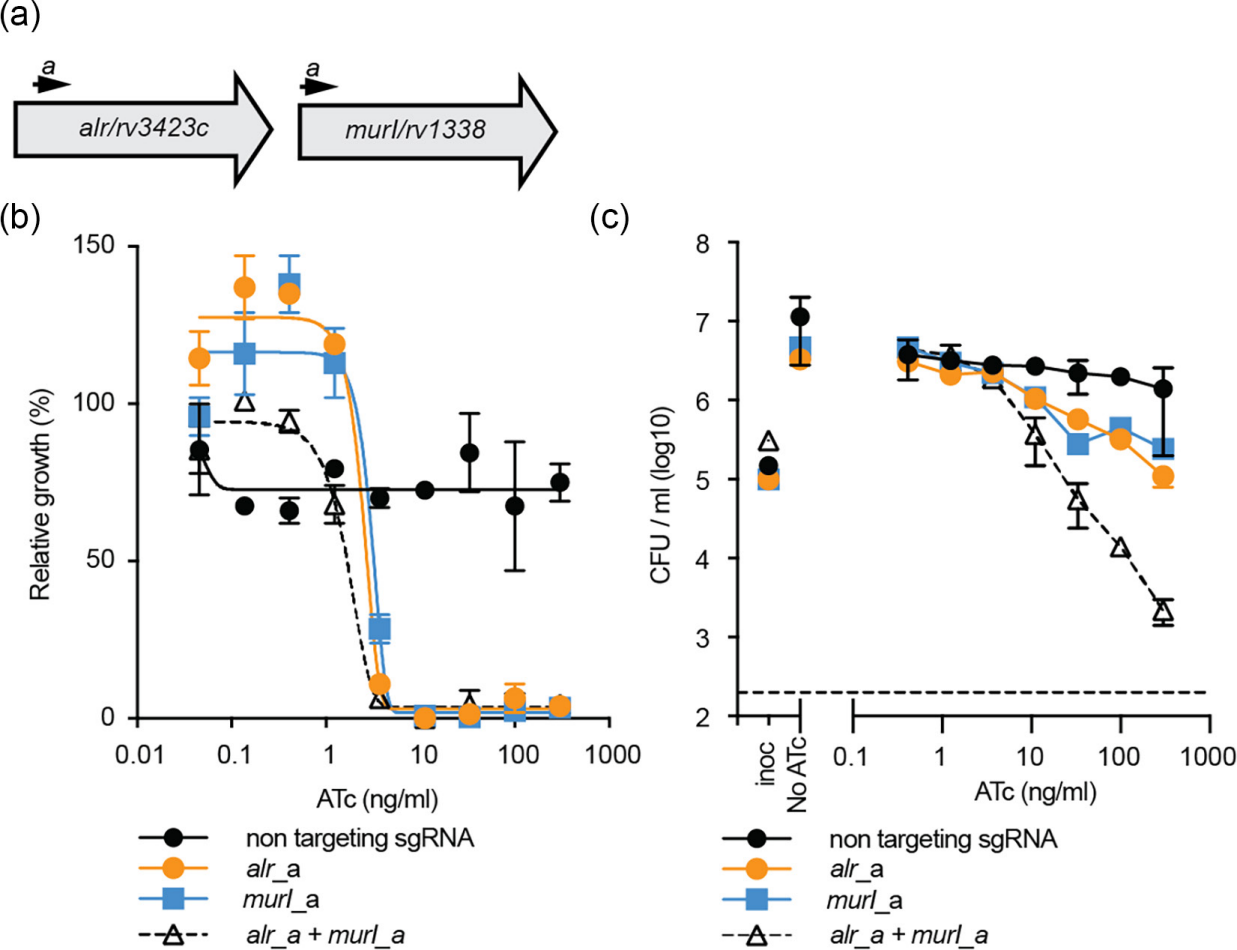
Transcriptional repression of *alr* and *murI* is synergistic in *M. tuberculosis*. (**a**) Approximate binding locations of gRNA sequences targeting each gene in dual targeting CRISPRi plasmids. (**b**) Growth of *M. tuberculosis* expressing either a non-targeting, *alr, murI* or *alr* and *murI*. Cultures were grown in 96-well plates starting from an OD_600_ of 0.005 with differing levels of ATc. OD_600_ was measured on day 10. Results are the mean±range of two replicates from a representative experiment and expressed relative to a no ATc control. (**c**) Viability of *M. tuberculosis* expressing either a non-targeting, *alr, murI* or *alr* and *murI*. Cultures were grown in 96-well plates starting from an OD_600_ of 0.005 with differing levels of ATc. c.f.u. ml^−1^ was determined after 5 days of growth and is presented as the mean±range of two replicates. Inoc, inoculating c.f.u. ml^−1^ on day 0.

We next hypothesized that if the simultaneous repression of *alr* and *murI* had a synergistic interaction, then dual CRISPRi transcriptional repression would either reduce the amount ATc needed to inhibit bacterial growth or alternatively have a lethal interaction when inhibiting two bacteriostatic genes. To investigate this, we constructed CRISPRi plasmids that expressed two gRNA sequences that individually targeted *alr* or *murI* using golden gate cloning as previously described (Fig. 2a) [27]. In CRISPRi phenotypic assays, targeting both *alr* and *murI* did not change the concentration of ATc needed to inhibit bacterial growth (Fig. 2b), yet it did produce a bactericidal effect on viability (Fig. 2c). Specifically, the repression of both *alr* and *murI* reduces viability by approximately 2-log_10_ c.f.u. ml^−1^ at 300 ng ml^−1^ ATc compared to the individual bacteriostatic gRNAs that did not reduce viability (Fig. 2c). The bactericidal effect of dual *alr+murI* targeting was concentration dependent, with an approximately 0.5 and 1-log_10_ reduction in c.f.u. ml^−1^ at 33 and 100 ng ml^−1^ ATc (Fig. 2c). In conclusion, the dual repression of *alr* and *murI* has a synergistic interaction against bacterial viability in *M. tuberculosis*.

**Fig. 3.**
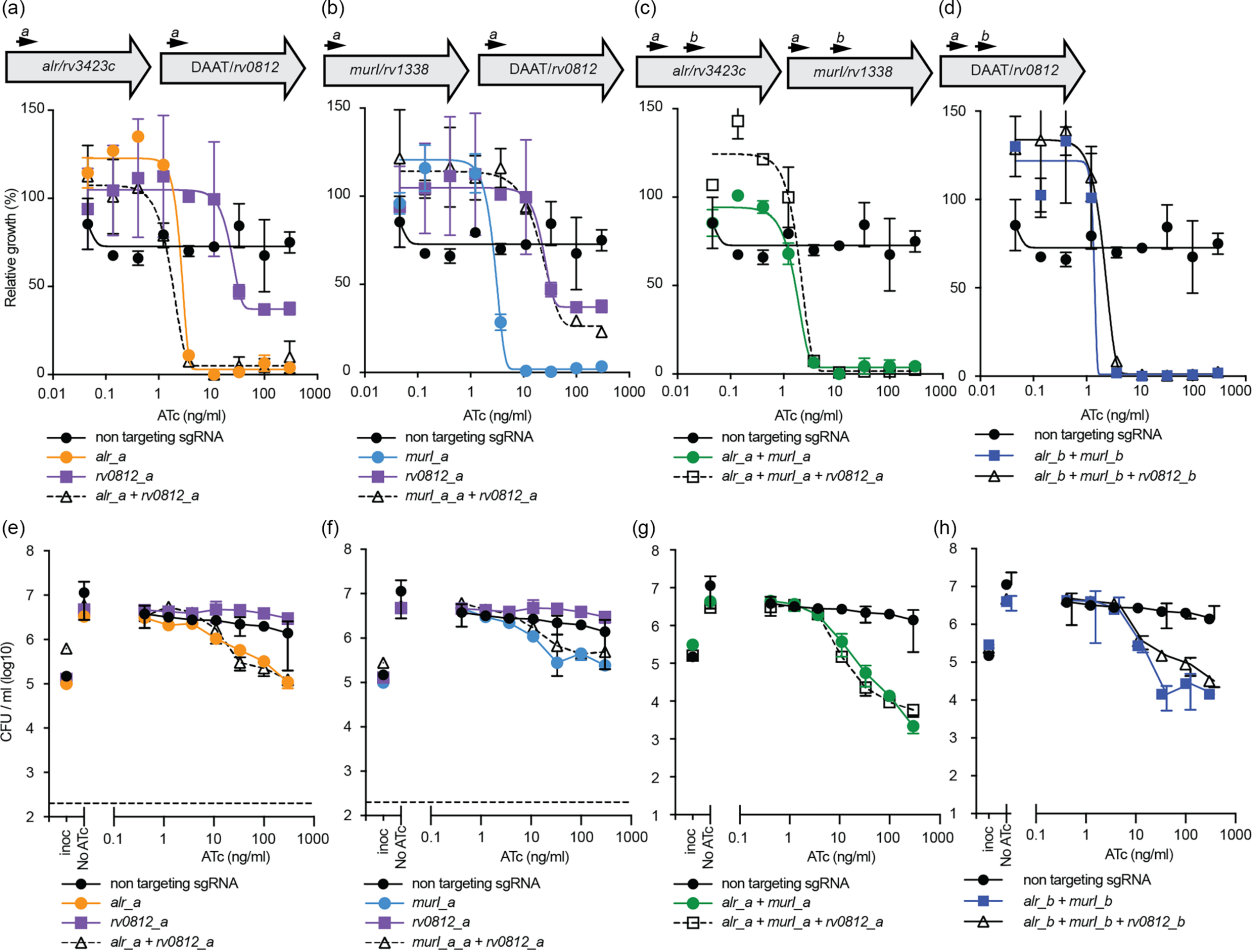
Transcriptional repression of *rv0812* does not interact with *alr* or *murI* repression that is synergistic in *M. tuberculosis*. (**a–d**) Approximate binding locations of gRNA sequences targeting each gene in multiplexed CRISPRi plasmids. (**a–h**) Growth of *M. tuberculosis* expressing either a non-targeting and (**a**) *alr*±*rv0812,* (**b**) *murI±rv0812* or (**c**) *alr+murI*±*rv0812*. Cultures were grown in 96-well plates starting from an OD_600_ of 0.005 with differing levels of ATc. OD_600_ was measured on day 10. Results are the mean±range of two replicates from a representative experiment and expressed relative to a no ATc control. (**d–f**) Viability of *M. tuberculosis* expressing either a non-targeting and (**d**) *alr*±*rv0812,* (**e**) *murI±rv0812* or (**f**) *alr+murI*±*rv0812*. Cultures were grown in 96-well plates starting from an OD_600_ of 0.005 with differing levels of ATc. c.f.u. ml^−1^ was determined after 5 days of growth and is presented as the mean±range of two replicates. Inoc, inoculating c.f.u.

We hypothesized that if *rv0812* was functionally redundant for *murI* activity in *M. tuberculosis*, then repression *rv0812* would have a synergistic interaction with either (i) repression of *alr* or (ii) repression of *murI* or would enhance the synergistic interaction between the dual repression of *alr* and *murI*. To investigate this, we constructed CRISPRi plasmids that expressed combinations of gRNA to repress either (i) *alr+rv0812*, (ii) *murI+rv0812* or (iii) *alr+murI+rv0812* using golden gate cloning as previously described (Fig. 3a–c) [27]. In CRISPRi phenotypic assays, the simultaneous targeting of *rv0812* with *alr* or *murI* did not change the concentration of ATc needed to inhibit bacterial growth (Fig. 3a–c). The repression of *rv0812* with either *alr* or *murI* also maintained the bacteriostatic effect on viability that was similar to the repression of *alr* or *murI* alone (Fig. 3d, e). We did note an apparent rescue, by CRISPR inhibition of *rv0812*, of some of the murI-induced growth defects. However, the repression of *rv0812* did not alter the bactericidal effect of the dual repression of *alr+murI* with the triple- and double-repression strain having similar effects on viability when using either the a or b gRNA (Fig. 3c, f). Prior work in *M. smegmatis* observed that mutations in the MSMEG_5795 (i.e. *rv0812* homologue) promoter were selected and upregulated MSMEG_5975 when a ∆*murI* mutant was grown for prolonged periods in the absence of d-Glu [16]. It is possible that in our experimental setup, the expression levels of *rv0812* in *M. tuberculosis* are too low and do not allow for rescue of *murI* transcriptional inhibition. In conclusion, the repression of *rv0812* does not augment the effect of inhibiting either *alr* or *murI* expression in *M. tuberculosis*.

Here, using CRISPRi transcriptional repression, we have investigated the interactions between *murI* and *alr* enzymes that contribute d-glutamate and d-alanine, respectively, to PG biosynthesis in *M. tuberculosis*. The simultaneous inhibition of both *murI* and *alr* had a synergistic interaction with regard to bacterial killing. Whilst we did not observe any reduction in the ATc concentration needed to inhibit growth, our observation of a synergistic killing interaction between *murI* and *alr* supports prior results showing an interaction between BCDA and DCS, chemical inhibitors of these respective enzymes [9]. Notably, *rv0812* did not appear to be functionally redundant for *murI*, within the parameters of our experimental paradigm. Given that DCS also targets Ddl, which codes for the essential enzyme Ddl, future work using CRISPR technology to explore synergy with this target would be of interest.

Although our results support the synergistic interaction between *murI* and *alr* inhibition, there are distinct differences between transcriptional and chemical inhibition. For example, CRISPRi transcriptional inhibition leads to the removal of the resulting protein and any other co-transcribed genes, whilst chemical inhibition only affects enzyme function leaving the protein largely intact. These differences may confound a direct correlation between our genetic study and future chemical interaction studies on target *murI* and *alr* inhibitors. In conclusion, these results highlight the potential of drug combinations that target *murI* and *alr* in addition to those that target multiple enzymes within cell wall biosynthesis.

## Abbreviations

ADCL: aminodeoxychorismate lyase
Alr: alanine racemase
ATc: anhydrotetracycline
BCDA: β-chloro-d-alanine
CRISPRi: CRISPR interference
DCS: d-cycloserine
gRNA: guide RNA
PDIM: phthiocerol dimycocerosate
PG: peptidoglycan
